# Composition and distribution of vegetation in the water level fluctuating zone of the Lantsang cascade reservoir system using UAV multispectral imagery

**DOI:** 10.1371/journal.pone.0247682

**Published:** 2021-03-29

**Authors:** Weiwei Jiang, Lun Liu, Henglin Xiao, Song Zhu, Wentao Li, Ying Liu

**Affiliations:** School of Civil Engineering, Architecture and Environment, Hubei University of Technology, Wuhan, Hubei Province, P.R.China; Universidade Federal de Uberlandia, BRAZIL

## Abstract

With the development of a large number of tall dams and large cascade reservoir projects in the Lantsang River Basin, a large water level fluctuating zone (WLFZ) containing cascading reservoirs has formed. This newborn ecosystem is related to the sustainable development of hydropower projects, and has become a new problem to be studied urgently. Taking WLFZs in the Huangdeng, Xiaowan and Nuozhadu Reservoirs in the Lantsang River Basin as study areas, this study used multi-spectral remote-sensing field data obtained with unmanned aerial vehicles (UAVs) to ascertain the species types, coverage, distribution characteristics, dominant species and pioneer species of naturally restored vegetation. The considered data were subjected to UAV data processing, vegetation classification using multi-spectral images and a geographic information system (GIS) terrain-distribution analysis. Results show that: Polygonum Plebeium, Cynodon dactylon, Xanthium sibiricum, Ageratum conyzoides, Eleusine indica, Digitaria sanguinalis and Verbena officinalis are the dominant species of vegetation that could be naturally restored in the WLFZ; the vegetation coverage and the number of species are significantly positively correlated with the age and restoration periods of the WLFZ; the vegetation coverage of each study area increases at first, and then decreases, as a function of elevation; gentle slopes about 0–25°are more suitable for vegetation restoration. This study provides first-hand data on the natural restoration of vegetation in WLFZs, and gives a useful reference for its ecological restoration as a consequence of hydropower cascade development in the Lantsang River Basin. Finally, the study demonstrates that light UAV remote sensing is an attractive choice for investigating vegetation in reservoir WLFZs.

## Introduction

With the increase in the number of large reservoirs around the world [1, 2], reservoir water level fluctuating zones (WLFZs), which are a new and fragile ecosystem, have attracted the attention of researchers [3, 4]. A WLFZ refers to a transitional area with alternating changes of water and land formed around a reservoir bank due to changing reservoir water levels, that is, the zone between the highest water level and the lowest water level [5–7]. Compared with natural riparian zones, WLFZs usually experience larger rises and drops in water levels due to their high water-storage capacities; hence, they are more severely impacted by alternating dry and wet periods, and their ecosystems are fragile [8]. During the early stages of dam construction and impoundment, any original vegetation in the WLFZ is flooded and subsequently disappears, and the WLFZ becomes exposed over a large area. The structure, functional status and ecological restoration of WLFZ ecosystems are all new issues that need to be solved, and they are related to the ecological security and sustainable development of hydropower systems around reservoir banks.

Vegetation is an important topic in the research and practice of WLFZ ecosystem management. The bibliometric literature study by Lu et al. showed that a total of 478 papers on the WLFZs of the Three Gorges Reservoir have been published in China and other countries from 1989 to 2013, of which 151 were related to vegetation, accounting for 31.59% [9]. Around the vegetation in the WLFZ, the related research is mainly focused on two aspects. One is from the aspects of plant physiology and ecology, including plant physiological characteristics, growth response, ventilatory tissue, photosynthetic response, antioxidant enzyme activity, non-structural carbohydrates and so on [10–13]. On the other hand, from the aspects of vegetation community composition, including flora, vegetation status, soil seed bank, species diversity, community structure, landscape pattern, spatial distribution, vegetation cover, succession trend and so on [14–18].

Existing research regarding WLFZ vegetation has gradually advanced from basic investigations to identifying the relevant mechanisms and application levels. And most studies mainly focused on single-stage reservoirs. However, in the Lantsang River Basin, a large WLFZ area has formed owing to hydropower cascade development [19–21], and consequently, data and information support are urgently required for degraded vegetation restoration. But at present, there is still a lack of basic investigations in the WLFZs of cascade reservoirs in the Lantsang River Basin.

In addition, the most commonly used investigation techniques for WLFZs, such as spot and quadrant surveys, are restricted by the precipitous topography of the studied reservoir, which leads to many places that are unreachable, and hence data acquisition is difficult, time-consuming and laborious, and the coverage is small. Satellite remote sensing has been tried in several studies of vegetation in WLFZs. Cui simulated the dynamic evolution process of vegetation coverage in the WLFZ of Guanting Reservoir based on seven-phase Landsat TM/ETM+/OLI remote sensing images [22]. Jiang et al. analyzed the characteristic of vegetation recovery in WLFZ of Three Gorges reservoir using multi-temporal GF-2 satellite images [23]. Yin et al. studied the 17-year dynamic changes in the WLFZ of Danjiangkou Reservoir using Landsat series and HJ-1A/B satellite images from 2000 to 2016 [24]. However, the limited spatial resolution of the data is not enough to support the study of vegetation at the species level. Instead, unmanned aerial vehicle (UAV) remote sensing is a very attractive alternative [25–27]. The centimeter-resolution of UAV remote-sensing data makes it possible to document any intra-microplot variability of vegetation species.

Therefore, in this study, we used UAV multi-spectral remote sensing to investigate vegetation in the WLFZs of the Lantsang River Basin, and obtain the species type, coverage, distribution characteristics, dominant species and pioneer species of the vegetation therein. Using these data, we analyzed the characteristics of the natural restoration properties of the vegetation, to provide some necessary information and theoretical guidance for the ecological restoration of the WLFZ of Lantsang River Reservoir.

## Materials and methods

### Study area

Lantsang River is one of the major rivers in southwest China, and the seventh longest river in the world. It originates from Jifu Mountain, Zaduo County, Yushu Tibetan Autonomous Prefecture, Qinghai Province. The total length of the main stream is 4909 km, of which the domestic length is 2139 km. The Lantsang River Basin has a southwest monsoon climate, with distinct dry and wet seasons. Generally, May to October is the wet season and November to April is the dry season. Approximately 85% of the precipitation is concentrated in the wet season, with June to August receiving the most. The hills and valleys of the basin are interlaced, and the temperature increases gradually from north to south, with subtropical or tropical climates. The average temperature is 15–22°C, the hottest average temperature is 20–28°C, the coldest average monthly temperature is 5–20°C and the annual precipitation is 1000–3000 mm. The amount of precipitation received in the valleys is less than that in the mountainous areas.

**Fig 1:**
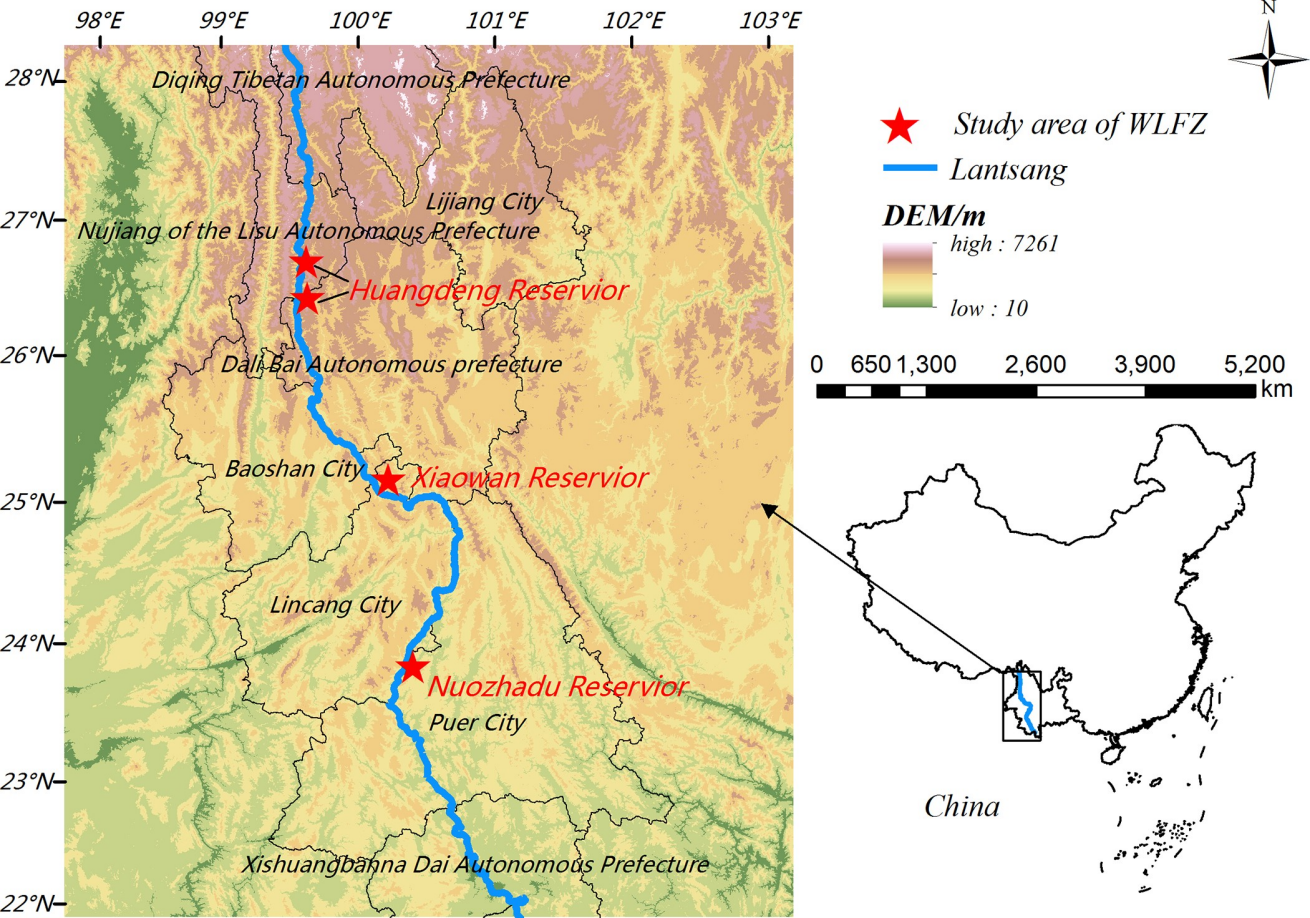
Location of the WLFZ study area (The SRTM 90m DEM map contained is provided by Geospatial Data Cloud site, Computer Network Information Center, Chinese Academy of Sciences. (http://www.gscloud.cn).

In this study, four WLFZs of three reservoirs were selected as study areas along the Lantsang River, including Huangdeng Hydropower Station, Xiaowan Hydropower Station and Nuozhadu Hydropower Station, as shown in Fig 1. The vegetation structure of the WLFZ in each study area is simple, all are herbaceous plants, mainly 1–3 dominant species, can be distributed in patches, the remaining species are usually less than 5, each species is only a few.

Huangdeng Reservoir is located in Lanping County, Yunnan Province, and it is the fifth step in the planning of the section from Gushui to Miaowei in the upper reaches of Lantsang River. The river valley, which is a type of alpine canyon, is narrow and V-shaped, with basically symmetrical topography, steep hillsides, a 40–70 m wide river surface with fast currents and a large water level drop. The normal water level elevation (altitude above sea-level, the same below) of the reservoir is 1619 m, and the reservoir has seasonal regulation performance. On November 28, 2017, the diversion tunnel was sluice impounded. When the data were collected in July 2019, the reservoir had just experienced a cycle of water storage–normal water level–water discharge–dead water level, which was during the exposure period of the WLFZ.

Xiaowan Dam is the leading reservoir of the cascade development in the middle and lower reaches of Lantsang River. It is a water storage dam with a multi-year regulation capacity. The normal water level elevation is 1240 m above sea level, the dead water level elevation is 1166 m, the reservoir area is 189.10 km^2^, and the reservoir area is an alpine canyon landform. 2010 was the impoundment period of Xiaowan Dam, where the water level elevation in front of the dam rose from 988 m to 1240 m, and the flow rate dropped rapidly to 0.01 m/s. At the beginning of Xiaowan Reservoir operation in 2011, a static water environment in the reservoir area was initially established.

Nuozhadu Reservoir is located in a subtropical monsoon climate scenic spot, with an annual average temperature of 17.8–22.2°C, and an annual precipitation of 912–1546 mm. Nuozhadu Reservoir has a normal water level elevation of 812 m, a dead water level elevation of 765 m, and a return water length of about 210 km. On November 6, 2011, the sluice began to impound water. In July 2012, it was impounded to a dead-water-level elevation of 765 m, and on October 17, 2013, it was impounded to a normal-water-level elevation of 812 m.

### UAV data acquisition

In this paper, the data acquisition platform was based on a Parrot Sequoia multispectral camera mounted on a DJI Inspire 2, which is a four-rotor UAV. The Parrot Sequoia camera was equipped with four 1.2 Mpx single channel sensors to collect data in discrete spectral bands: green (wavelength = 550 mm; bandwidth = 40 nm), red (wavelength = 660 mm; bandwidth = 40 nm), red-edge (wavelength = 735mm; bandwidth = 10 nm), and near-infrared (wavelength = 790 mm; bandwidth 40 = nm), and the image size is 1280 × 960 pixels. The system also included a 16 Mpx RGB camera with an image size of 4608 × 3456 pixels.

The UAV data acquisition lasted 12 days, from July 6 to 17, 2019, with an average flight time of 2 h in each experimental area. The amount of forward and lateral overlap was greater than 80%, the camera shooting interval was set to 1.0 s, and the flight speed was less than 2 m/s. Considering the large fluctuations in the terrain in the experimental area, the altitude was controlled between 15–28 m.

Before flying, 8–10 image control points were evenly arranged in the study area. Considering reflection, the image control board was designed with a white smooth surface on one side, and a brown rough surface on the other. When the sun was strong, the white board may become overexposed quickly, and the control point marks may not be recognized; in this case, a brown board was used. In instances when the sun was not overly bright, a whiteboard was used to provide sufficient reflection so that the control point marks could be clearly imaged. The size of each board was 60 × 60 cm, and the control points were represented by red diagonal intersections, with numbers attached. The longitude, latitude and elevation of the control points were measured based on a CORS station using K9 series GPS (static plane accuracy: ± 3 mm + 1 ppm, static elevation accuracy: ± 5 mm + 1 ppm).

**Fig 2:**
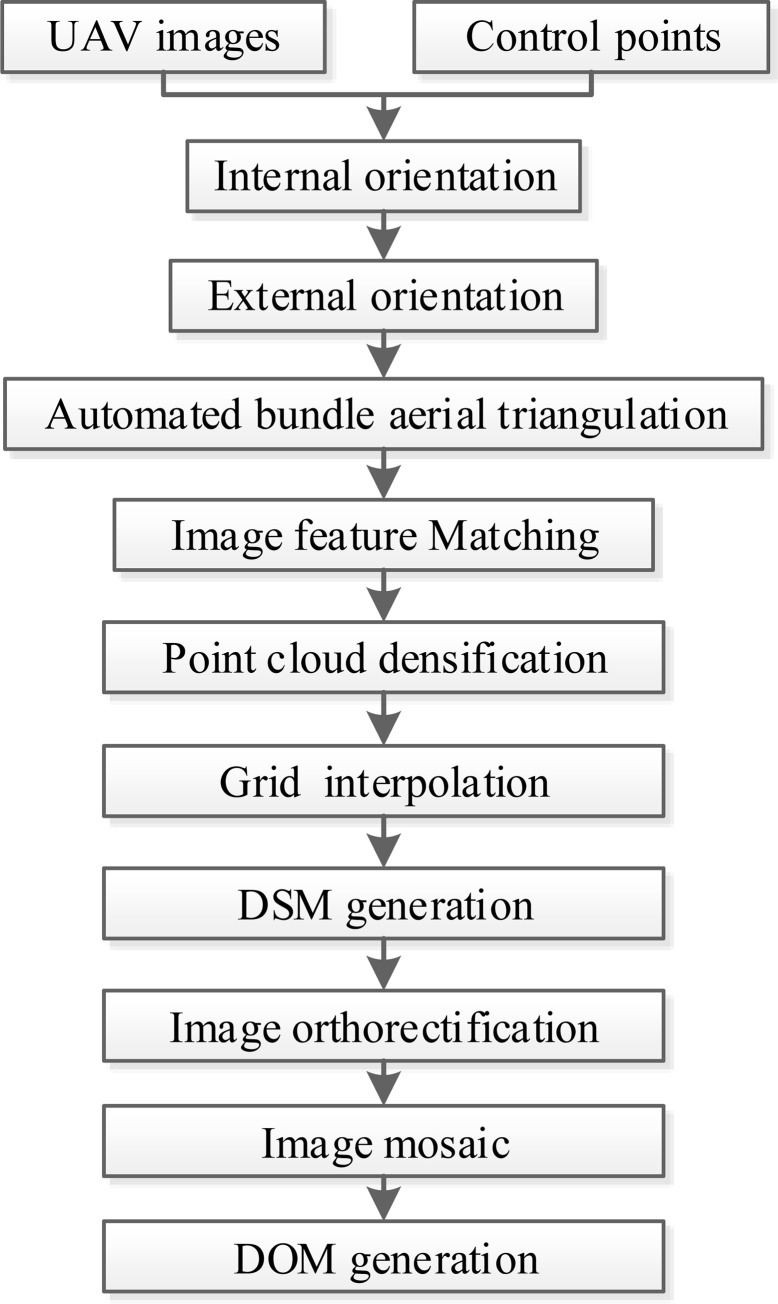
The workflow of photogrammetric processing based on Pix4dmapper software.

**Fig 3:**
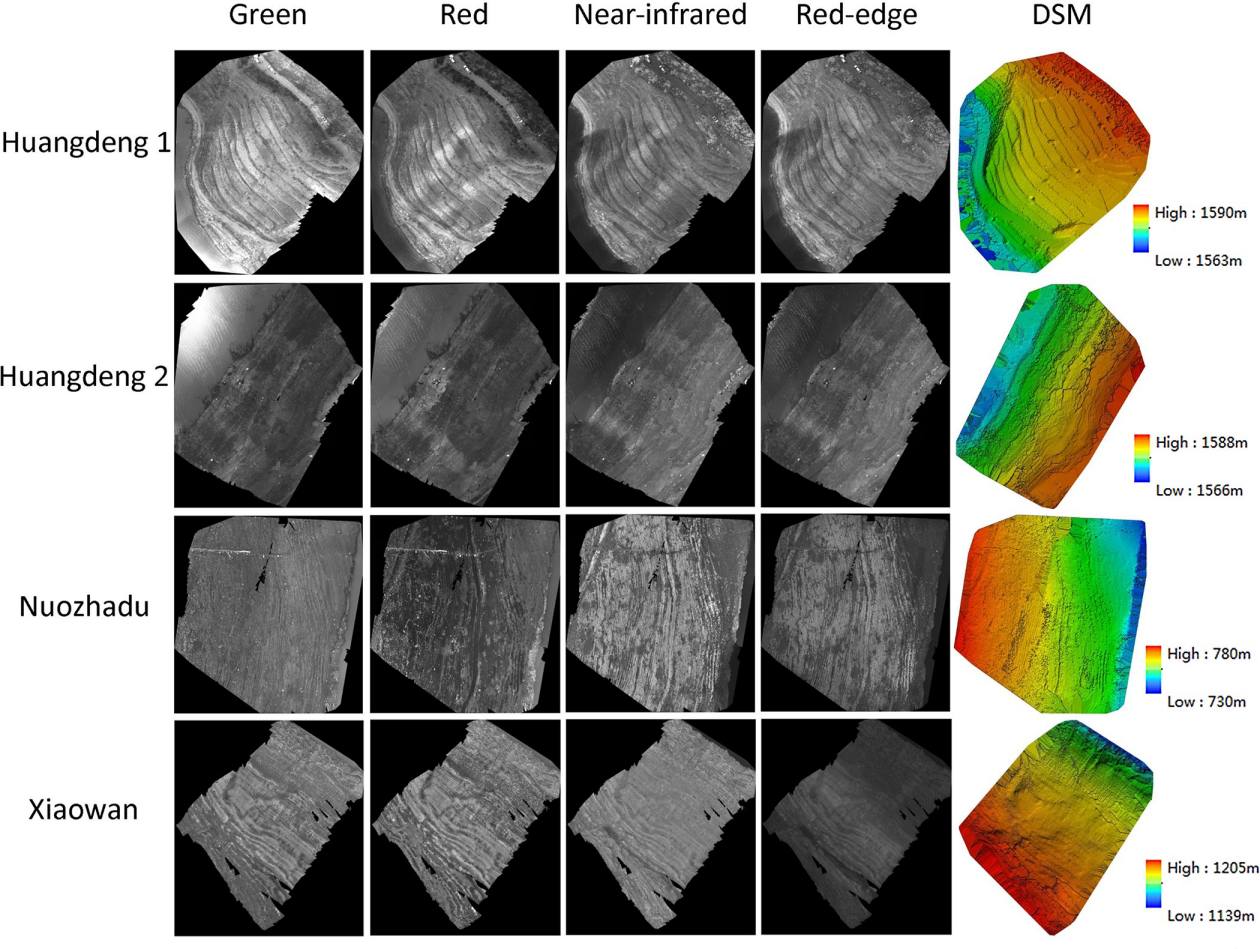
The digital orthophoto maps and digital surface models of the study areas.

For the obtained UAV images and control points, the auto-workflow of photogrammetric processing based on Pix4dmapper software (Fig 2) was carried out for each UAV block to generate the digital orthophoto map (DOM) and digital surface model (DSM) data of the research area (Fig 3). [28]. According to the result reports of Pix4dmapper, the average ground sampling distances of DOMs of Huangdeng1, Huangdeng2, Nuozhadu and Xiaowan study area are 2.10cm, 1.18cm, 1.36cm and 1.19cm respectively.

### Field data acquisition

**Fig 4:**
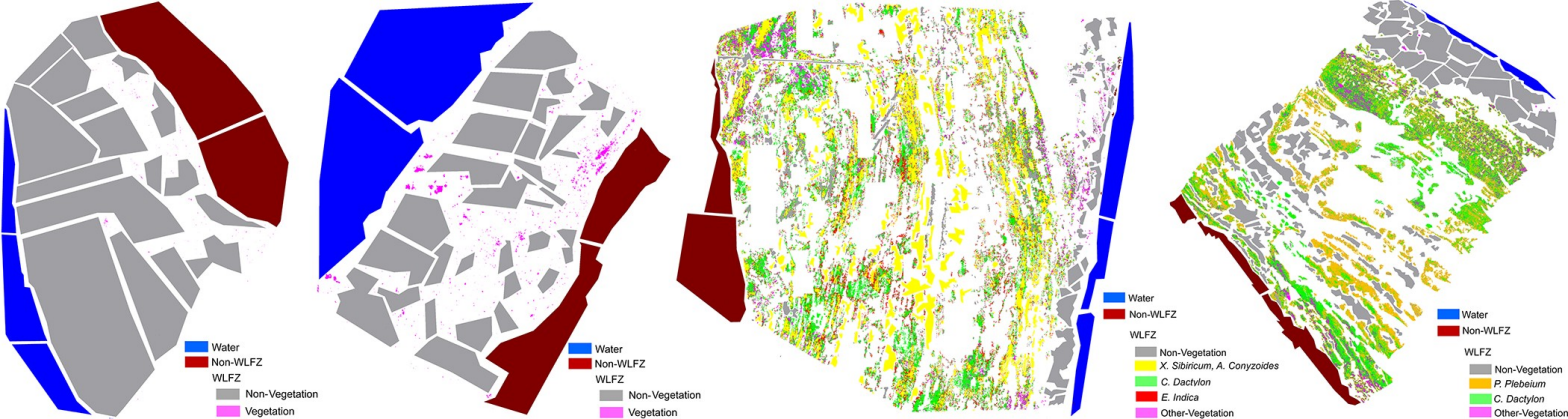
The ground truth reference of (a) Huangdeng 1, (b) Huangdeng 2, (c) Nuozhadu and (d) Xiaowan study area.

After each air flight was completed in each research area, field investigations were conducted. These investigations mainly included recording the composition and distribution of vegetation species, identifying the dominant species, taking pictures of plants and terrain in the WLFZ, analyzing the boundary line of the WLFZ and taking images according to the watered-out traces of the shore features, so as to collect interpretation marks for the remote-sensing images of the WLFZ. The vegetation types of each study area in the WLFZs are shown in Table 1. According to field investigation, samples of each category were randomly collected through visual interpretation of DOM to obtain ground truth references of each study area, as shown in Fig 4.

**Table 1:** Vegetation types of each study area.

Study area	Vegetation type
Huangdeng 1	*C*. *Dactylon*
Huangdeng 2	*D*. *Sanguinalis*, *V*. *Officinalis*
Nuozhadu	*X*. *Sibiricum*, *A*. *Conyzoides*, *C*. *Dactylon*, *E*. *Indica*, *Rorippa indica*, *Equisetum hyemale*, *P*. *Plebeium*
Xiaowan	*C*. *Dactylon*, *P*. *Plebeium*, *Salvia plebeia*, *Chenopodium ambrosioides*, *Bidens bipinnata*, *X*. *Sibiricum*, *A*. *Conyzoides*, *Calystegia hederacea*

### Vegetation mapping of WLFZ

Using eCognition as the algorithm platform, the DSM and DOM data of the near-infrared, red, green and red-edge bands were taken as the input layer, and the normalized difference vegetation index (NDVI) was calculated as an additional layer. Based on the object-oriented idea, the fractal net evolution approach (FNEA) algorithm was used to segment the multi-spectral images to obtain segmentation objects, in which the near-infrared band, red-edge band and NDVI layer, which are closely related to vegetation, were selected to participate in the segmentation calculations [29]. Visual interpretation is usually the most meaningful method for parameter segmentation in natural environments [30]; thus, the optimal image segmentation parameters were set by parameter combinations determined via trial-and-error and visual interpretation, the segmentation scale, shape and compactness parameters of Huangdeng1, Huangdeng2, Nuozhadu and Xiaowan study area are (20, 0.4, 0.5), (50, 0.4, 0.5), (90, 0.4, 0.5), and (50, 0.4, 0.5) respectively. It is difficult to avoid some segmentation errors [31]; hence, for some undersegmented vegetation objects, manual intervention and separation was employed to avoid confusion between vegetation and other land types, as well as distinguishing between the different types of vegetation.

**Fig 5:**
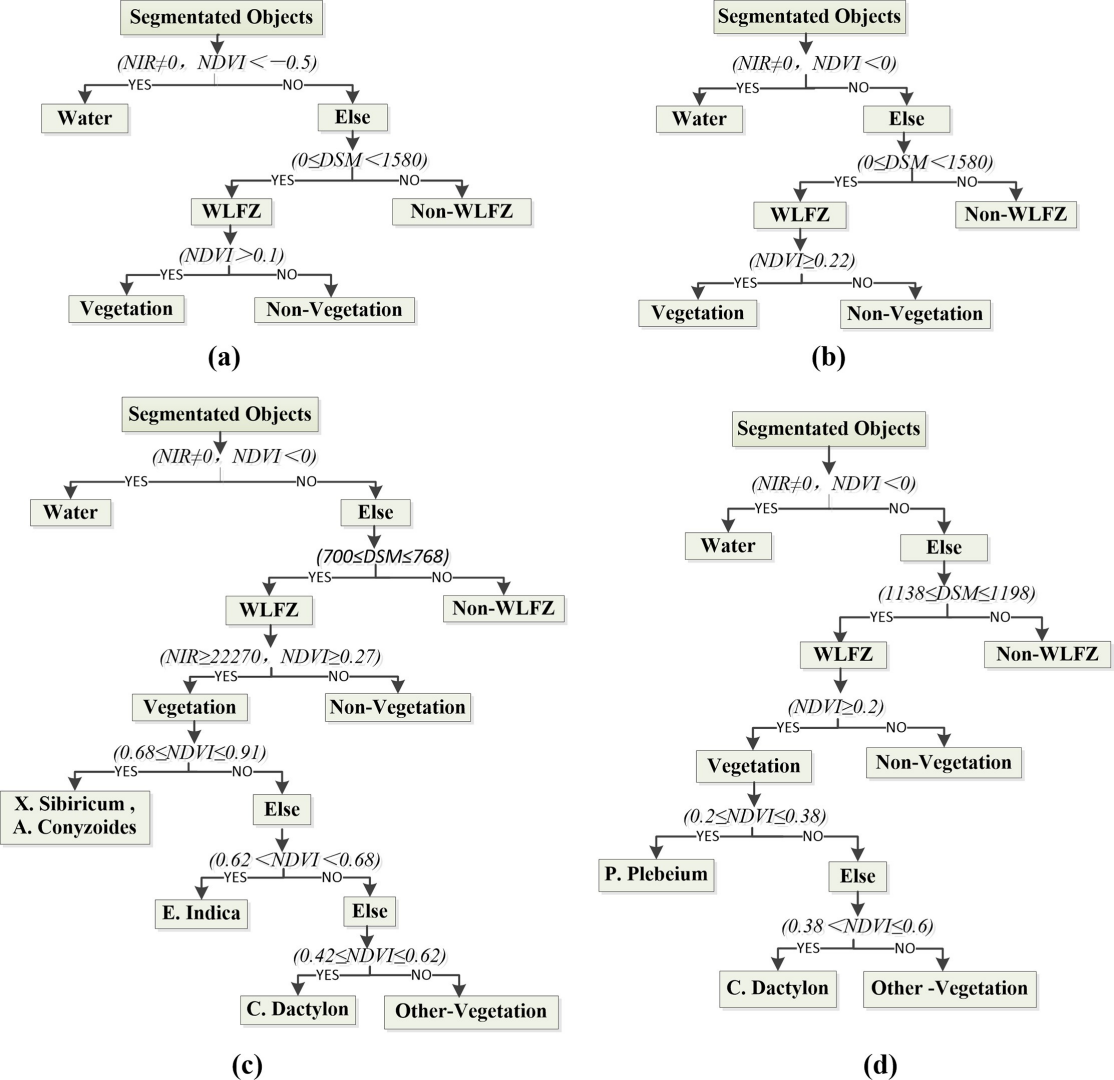
The rule-based classification tree of (a) Huangdeng 1, (b) Huangdeng 2, (c) Nuozhadu and (d) Xiaowan study area.

After the segmentation process was completed, the vegetation in the WLFZs was classified by the rule-based hierarchical classification method and visual interpretation. A rule-based method was implemented using a classification tree, where each node was divided into two branches according to the chosen rules [32, 33]. This method has been demonstrated to be both flexible and robust [34]. The NDVI, DSM and spectral average of each band were mainly adopted in the classification features. The rule-based classification tree of each study area is shown in Fig 5. The whole classification process was divided into two parts. In the first part, the whole study area was classified into 3 categories of water, WLFZ and Non-WLFZ with hierarchical rule layers; in the second part, the extracted WLFZ was divided into vegetation and Non-vegetation, then the vegetation may further classified if the subclasses can be identified. While the spectral differences of the different vegetation categories were used to construct the rules to obtain vegetation subclasses, which were combined with the results of the visual interpretation to manually modify the classification results. The classification results were evaluated by producer accuracy, user accuracy, overall accuracy and kappa coefficient as compared with ground truth references.

### Analysis of vegetation distribution characteristics

Topographic features are an important indicator of habitat conditions [35], which directly affect the spatial distribution and population pattern of vegetation. Elevation, slope and aspect are important indices used to describe topography, which are closely related to changes in vegetation. Slope affects surface runoff, soil erosion and soil water holding capacity, while aspect changes determine the intensity of light reaching the ground, which affects vegetation growth and development. The topography of the Lantsang River Basin is steep, and the elevation changes considerably. The flooding time, frequency, light intensity and heat conditions at different elevations in the WLFZ are significantly different. In addition, the WLFZ is greatly affected by water bodies; so, the nearest distance to surrounding water bodies was also used as an additional topographic element.

Because the cover types of the WLFZ are mainly bare soil, low herbaceous vegetation and stones, and there are no tall features, the slope aspect, slope and distance maps to the nearest water body were calculated based on the DSM with ArcGIS software. Then, with a spatial overlay analysis of vegetation classification maps and terrain data, the spatial distribution of vegetation can be provided in an accurate and quantitative way. Among them, the slope is mainly referred to the “classification standard for the risk of soil and water loss” (sl718-2015) issued by the Ministry of Water Resources of the People’s Republic of China, which is divided into five grades: micro degree (0–8°), light degree (8–15°), medium degree (15–25°), heavy degree (25–35°), and extreme degree (>35°). Aspect was determined according to nine directions: east, south, west, north, northeast, southeast, northwest, southwest and plane. Elevation and the nearest water body distance were divided according to personal experience into numerical ranges.

Natural restoration of vegetation is a long process [36–40], especially in WLFZs due to frequent flooding. As each cascade reservoir is quite different in terms of its age, the cumulative growth period and the succession stages of the vegetation in the WLFZ of each reservoir are different. Therefore, these time characteristics of the vegetation were analyzed according to the age of each reservoir.

## Results

### Areal extent, composition and distribution of vegetation species in the WLFZs

**Fig 6:**
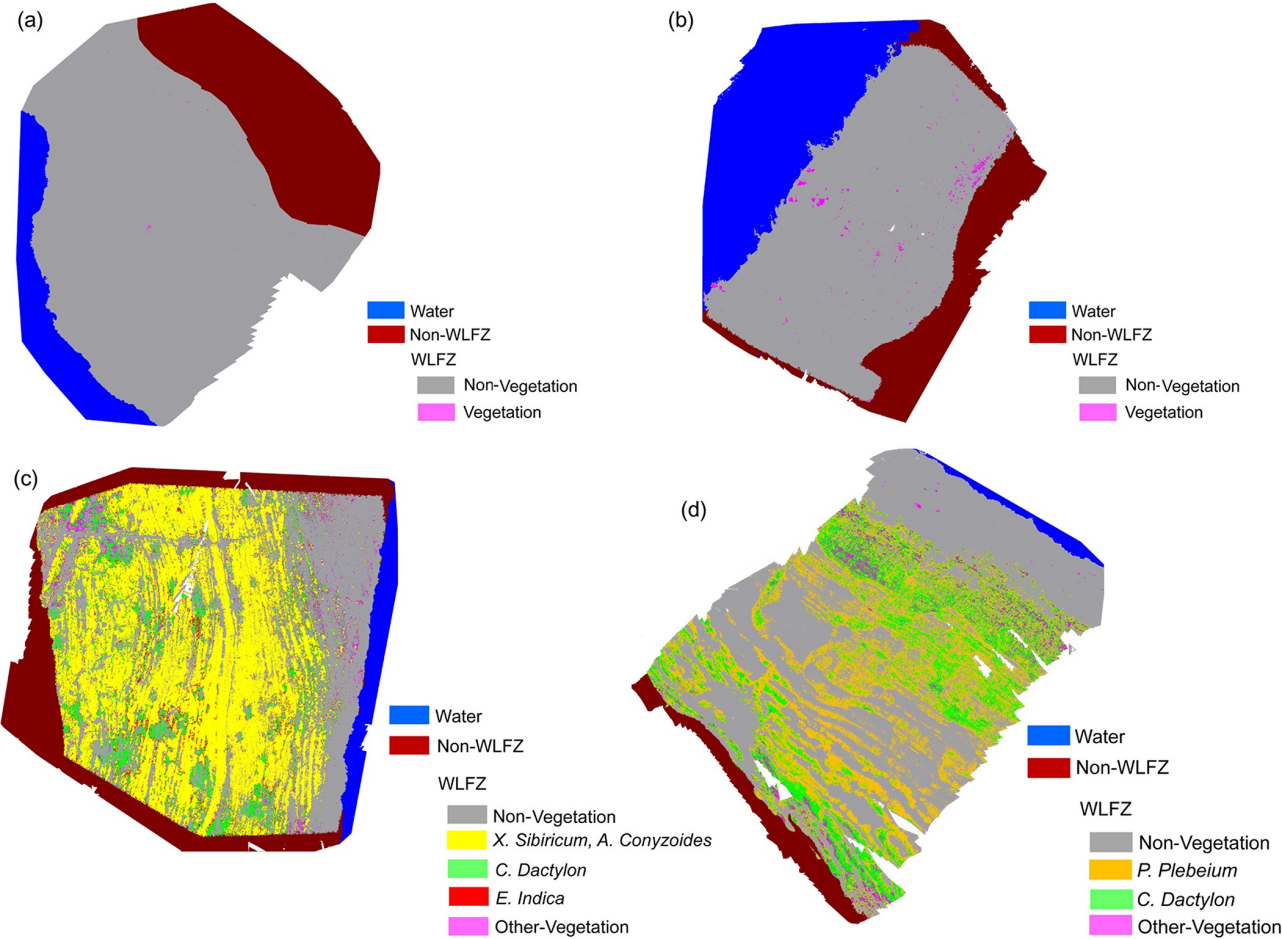
Classification results in the WLFZ of (a) Huangdeng study area 1, (b) Huangdeng study area 2, the (c) Nuozhadu study area, and the (d) Xiaowan study area.

There are two study areas in the WLFZ of Huangdeng Reservoir area. The first (Huangdeng 1) is remote and inaccessible. The topography is relatively flat, the vegetation type is single, and there is only *C*. *Dactylon*. In the second study area (Huangdeng 2), there is a mud path leading to the WLFZ, where nearby residents often fish, human activity is relatively common, the terrain is relatively steep, and the vegetation types are *D*. *Sanguinalis* and *V*. *Officinalis*. The classification results of the two study areas are shown in Fig 6(A) and 6(B) and Table 2, respectively. According to the classification results, the area of the WLFZ in the first study area is larger, up to 7853.44 m^2^, and the vegetation coverage rate is 0.05%; the WLFZ in the second study area is relatively small, 1470.72 m^2^, and the vegetation coverage rate is 1.08%.

**Table 2:** Vegetation area, percentage and classification accuracy of WLFZs in each study area.

Study area	Category		Area(㎡)	Percentage		Accuracy			
						user	producer	OA	KIA
Huangdeng1	Non-Veg		7849.83	99.95%		98.8%	95.5%	95.8%	0.905
	Veg		3.61	0.05%		93.1%	93.1%		
Huangdeng2	Non-Veg		1454.81	98.92%		98.1%	96.5%	97.0%	0.950
	Veg		15.91	1.08%		97.3%	100%		
Nuozhadu	Non-Veg		3067.43	37.56%		93.9%	98.8%	94.1%	0.912
	Veg	*X*. *Sib*, *A*. *Con*	*3825*.*46*	*75*.*03%*	62.44%	*94%*	*99*.*5%*		
		*C*. *Dac*	*735*.*57*	*14*.*43%*		*96*.*9%*	*81%*		
		*E*. *Ind*	*343*.*42*	*6*.*74%*		*95*.*6%*	*91*.*2%*		
		*Other-Veg*	*194*.*4*	*3*.*81%*		*90*.*7%*	*92*.*6%*		
Xiaowan	Non-Veg		9895.38	54.5%		99.1%	100%	98.7%	0.981
	Veg	*P*. *Ple*	*4870*.*90*	*58*.*96%*	45.50%	97.8%	99.4%		
		*C*. *Dac*	*2797*.*60*	*33*.*86%*		98.6%	96.1%		
		*Other-Veg*	*593*.*35*	*7*.*18%*		99.3%	93.6%		

*Note*: *X*. *Sib (X*. *Sibiricum)*, *A*.*Con (A*. *Conyzoides)*, *C*.*Dac (C*. *Dactylon)*, *E*.*Ind (E*. *Indica)*, *P*. *Ple (P*. *Plebeium)*, *C*.*Dac (C*. *Dactylon)*, Veg (Vegetation), Other-Veg (Other-Vegetation), Non-Veg (Non-Vegetation), OA (Overall Accuracy), KIA (kappa coefficient).

Generally, the vegetation coverage of the WLFZ in the Huangdeng Reservoir area is very low, and the species type is single; this may be related to its age. At the time of data collection in July 2019, the Huangdeng Reservoir area had just experienced the first cycle of high water storage–discharge–dead water level. Thus, the WLFZ had just formed, a large fraction of the primary vegetation was flooded and a large number of withered roots and residual branches could be seen at the scene. Thus, the collected data were “first-hand data” taken just after the formation of the WLFZ. It was observed that *C*. *Dactylon*, *D*. *Sanguinalis* and *V*. *Officinalis* are the pioneer species of vegetation restoration in the WLFZ, especially *C*. *Dactylon*. As the first study area is more than five times larger than the second study area, and there is less human interference, *C*. *Dactylon* is more likely to be the pioneer species of natural restoration. In the second study area, it can be seen from Fig 6(B) that large sections of vegetation are concentrated in the upper part of the WFLZ near the mud path, which may be affected by human activities. Indeed, human activity in the WLFZ may have a promoting effect on the propagation of vegetation seeds.

In the WLFZ of the Nuozhadu Reservoir area, there is large vegetation coverage, which is mainly composed of *X*. *Sibiricum* and *A*. *Conyzoides* associations, and there are some *C*. *Dactylon* and *E*. *Indica*. In addition, there are also several plants of *R*. *Indica*, *E*. *hyemale* and *P*. *Plebeium*. Therefore, the vegetation was divided into four types: *X*. *Sibiricum–A*. *Conyzoides*, *C*. *Dactylon*, and *E*. *Indica* and other vegetation, as shown in Fig 6(C) and Table 2.

Nuozhadu Reservoir completed its first high impoundment on October 17, 2013, i.e., more than five years earlier than Huangdeng Reservoir. Hence, the WLFZ has experienced more vegetation growth periods. Consequently, the vegetation has recovered well, with a coverage rate of 62.44%. Among them, *X*. *Sibiricum and A*. *Conyzoides* are both annual herbs with mixed growth, which have spread all over the WLFZ in the form of associations, thus accounting for 75.03% of the total vegetation; indeed, they are the dominant species. The second is *C*. *Dactylon*, with an area of 735.57 m^2^, accounting for 14.43% of the total vegetation, which grows throughout the entire WLFZ. Next, *E*. *Indica* accounts for 6.74%, which is mainly distributed in the middle of the WLFZ. The remaining vegetation, such as *R*. *Indica*, *E*. *hyemale and P*. *Plebeium*, are mostly distributed along the top and bottom of the WLFZ, accounting for 3.81%.

According to the field investigations, it was found that *P*. *Plebeium* and *C*. *Dactylon* are the main species of vegetation in the WLFZ of the Xiaowan Reservoir area. Although there are many other plant types, including *S*. *plebeia*, *Ch*. *Ambrosioides*, *B*. *Bipinnata*, *X*. *Sibiricum*, *A*. *Conyzoides* and *C*. *Hederacea*, each type is scarce in quantity, and the seedlings are small. Indeed, with the centimeter-scale resolution of the multispectral image vegetation classification data, such plants are difficult to identify, although the vegetation was further subdivided into *C*. *Dactylon*, *P*. *Plebeium* and other vegetation (Fig 6(D) and Table 2). The WLFZ has a large area of 18,157.23 m^2^, and its vegetation area is 8261.85 m^2^, accounting for 45.50%.

As mentioned previously, natural vegetation restoration takes time. The WLFZ of Xiaowan Reservoir initially formed in 2011, eight years before our study in July 2019. This may explain the restoration of its vegetation. The classification results further show that *P*. *Plebeium* is dominant in terms of vegetation restoration, accounting for 58.96% of the total vegetation area, which is widely distributed in all parts of the WLFZ, especially in the middle and upper parts, growing in patches. The second is *C*. *Dactylon*, accounting for 33.86%, which is mostly distributed in the top and middle of the WLFZ. The remaining 7.18% of the vegetation is *S*. *plebeia*, *Ch*. *Ambrosioides*, *B*. *Bipinnata*, *X*. *Sibiricum*, *A*. *Conyzoides and C*. *Hederacea*, among which *A*. *Conyzoides and C*. *Hederacea* are sparsely distributed along the top of the WLFZ, *B*. *Bipinnata* and *X*. *Sibiricum* mainly appear in the middle of the WLFZ, and *S*. *plebeia* and *Ch*. *Ambrosioides* are mostly found in the lower parts of the WLFZ.

### Topographical characteristics of the vegetation distributions in the WLFZs

#### Vegetation distribution as affected by elevation

**Fig 7:**
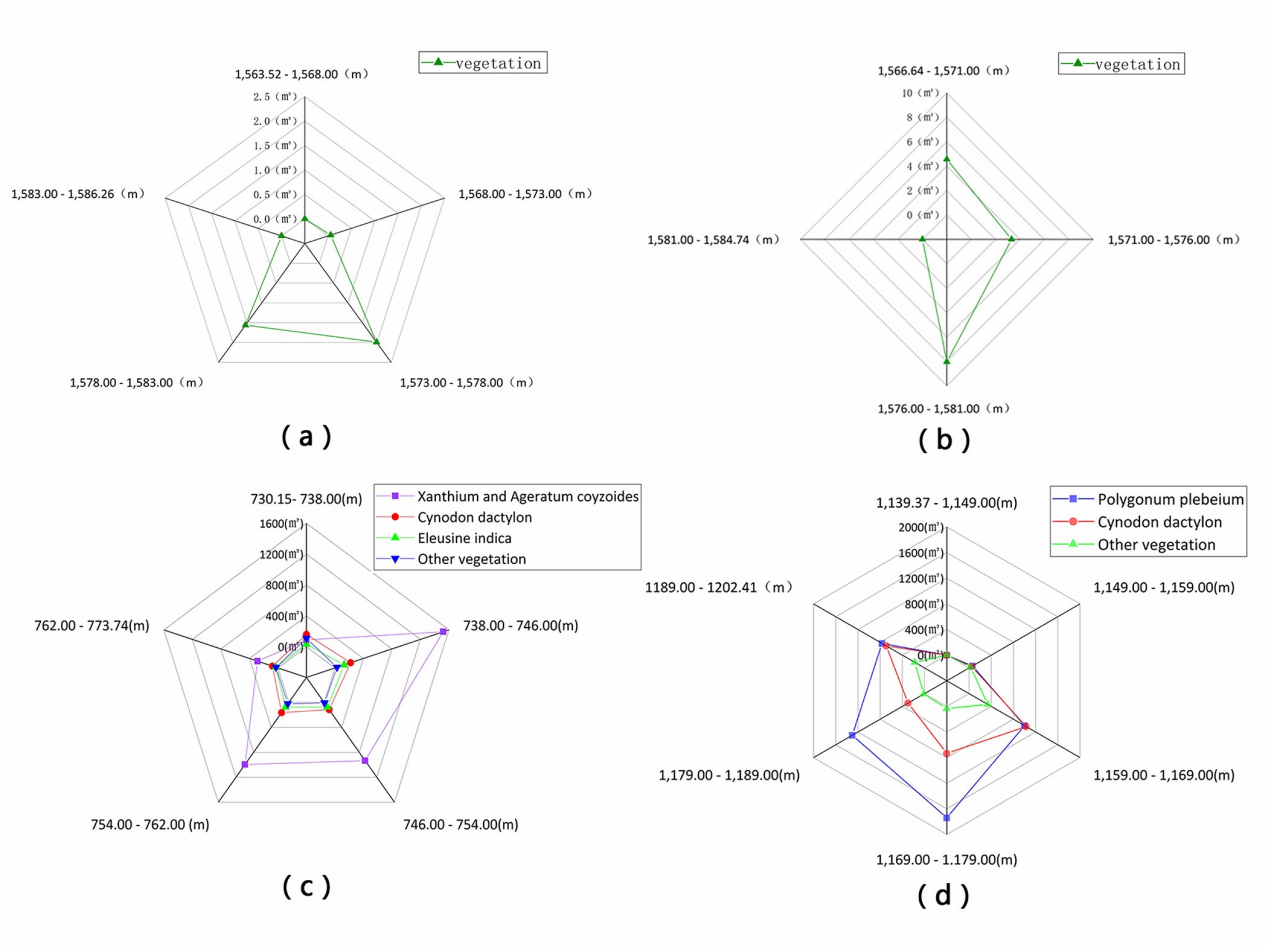
Vegetation distribution area as a function of elevation in the WLFZ of (a) Huangdeng study area 1, (b) Huangdeng study area 2, the (c) Nuozhadu study area, and the (d) Xiaowan study area.

As shown in Fig 7(A), 98.33% of the vegetation is distributed between elevations (ellipsoidal height measured by GPS, the same below) of 1573 to 1583 m in study area 1 of Huangdeng Reservoir, while vegetation in the areas below and above this elevation range is rare. The highest elevation range of the vegetation coverage in study area 2 is 1576–1581 m (Fig 7(B)), which is in the middle of the WLFZ.

In the Nuozhadu Reservoir study area (Fig 7(C)), 83.39% of the vegetation in the WLFZ is distributed between elevations of 738 to 762 m, while in the WLFZ above and below this elevation range, the vegetation is collectively less than 10%. In the different regions, except that the lowest part (elevation = 730–738 m), of the WLFZ, the vegetation is mainly *C*. *Dactylon* with some other plants, while the dominance of *X*. *Sibiricum–A*. *Conyzoides* clusters is obvious at other elevations, especially between 738 to 746 m, accounting for 76.84% of the total vegetation. In addition, the *X*. *Sibiricum–A*. *Conyzoides* groups are obviously affected by elevation, and their coverage rate sharply reduces outside the elevation range 738–762 m; similar distributions are seen for *E*. *Indica* and other vegetation. In contrast, the survival area of *C*. *Dactylon* is the same at all elevations, and its distribution is relatively uniform.

In Xiaowan Reservoir (Fig 7(D)), 78.56% of the vegetation in the WLFZ is distributed between elevations of 1159 to 1189 m, while 19.74% is distributed above an elevation of 1189 m, and only 1.7% is distributed below 1159 m. Among them, *P*. *Plebeium* is mainly distributed in the upper-middle regions of the WLFZ (1169–1189 m), *C*. *Dactylon* is more distributed in the lower-middle sections of the WLFZ (1159–1179 m), while other vegetation is mainly distributed in the lower-middle reaches of the WLFZ (1159–1169 m) and the uppermost part of the WLFZ (1189.00–1202.41 m). Between 1159 to 1169 m, there is no difference in the area of *P*. *Plebeium* and *C*. *Dactylon*, both accounting for 1000 m^2^. When the elevation increases by 10–20 m, *P*. *Plebeium* is more widely distribution, having about twice the area of *C*. *Dactylon*, while the other vegetation distributions decrease sharply. As the elevation increases further, the overall amount of vegetation decreases. At the top of the WLFZ, the amount of vegetation increases again, and the areas of *P*. *Plebeium* and *C*. *Dactylon* tend to be approximately equal. Generally, at all elevations, *P*. *Plebeium* and *C*. *Dactylon* are the dominant species in Xiaowan’s WLFZ, while the amount and variety of other species are mixed. Indeed, the vegetation in the WLFZ shows diversity, and the vegetation coverage area is considerable.

#### Vegetation distribution as affected by the nearest water distance

**Fig 8:**
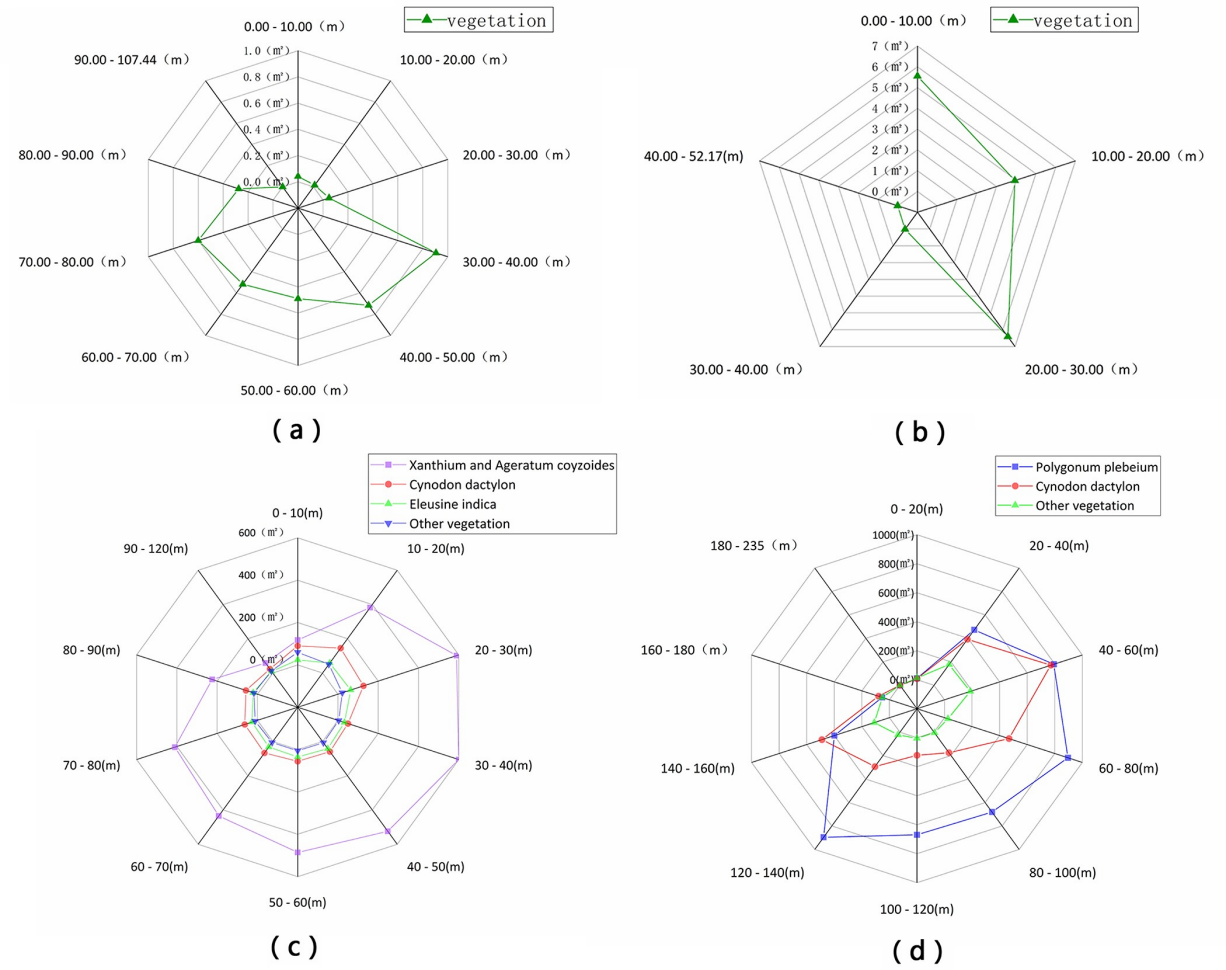
Vegetation distributions as a function of distance to the nearest water body in the WLFZ of (a) Huangdeng study area 1, (b) Huangdeng study area 2, the (c) Nuozhadu study area, and the (d) Xiaowan study area.

In study area 1 of Huangdeng Reservoir (Fig 8(A)), 89.43% of the vegetation in the WLFZ is distributed between 30 and 80 m away from the nearest water body, with less than 10% in the nearest (0–30 m) and farthest (90–107 m) sections. Compared with study area 1, the distance between the whole WLFZ and the nearest water body is different than in study area 2 (Fig 8(B)), where 99.89% of vegetation is located within 30 m of a water body in the WLFZ, while the vegetation in the further zones amounts to only 0.11%.

In the WLFZ of the Nuozhadu Reservoir (Fig 8(C)), *X*. *Sibiricum–A*. *Conyzoides* clusters are the dominant vegetation type in each nearest water body distance zone, 89.53% of which are distributed between 10 and 80 m from a water body, 5.87% between 80–90 m, and only 4.59% in the farthest (> 90 m) and the nearest (< 10 m) distance sections. *C*. *Dactylon* is distributed uniformly at all distance sections, slightly more at 10–30 m than other distance sections, but there was almost no survival at the farthest section (> 90 m). *E*. *Indica* is mainly distributed at distances less than 70 m, and although their quantity is small, they are evenly distributed. Other vegetation is mainly distributed between 0 and 20 m to the nearest water body, and the survival rates are close to 0 with increasing distance. Although the distribution preferences of the various vegetation types are different in each distance section, on the whole, the amount of vegetation closest to, and farthest from, the nearest water body is the least.

In Xiaowan Reservoir (Fig 8(D)), the most dominant species of *P*. *Plebeium* was found to have proportions of 80.80%, 9.70% and 8.20%, which are distributed from 40 to 140 m, 20 to 40 m, and 140 to 160 m, respectively, and almost none survive within 20 m and more than 180 m away from the nearest water body. 58.31% of *C*. *Dactylon* is distributed in the WLFZ between 20 to 80 m from the nearest water source, 28.01% in 120–160 m, 10.59% in 80–120 m, and 2.83% in 160–180 m. Proportions of 58.31%, 10.59% and 28.01% were found for *C*. *Dactylon* distributed from 20–80 m, 80–120 m, and 120–160 m distance ranges, respectively. Similarly, there is almost no survival in zones within 20 m, and more than 180 m away, from the nearest water body. 61.25% of the other vegetation is distributed in the 20–60 m distance section, while the remaining 27.18% is distributed from 140 to 180 m; distributions at other distances tend to be zero. On the whole, the number of P. Plebeium is absolutely dominant in the 60–140 m distance section; at distances of 40–60 m and 140–160 m, the numbers of C. Dactylon and P. Plebeium are equal to, or greater than, that of P. Plebeium; vegetation restoration is difficult within 20 m and more than 180 m away from the nearest water body.

According to the four study areas, the coverage of all kinds of vegetation in the furthest section of the WLFZ from the nearest water body is extremely low, and most of the vegetation grows in the middle distance section; the lowest occurrence of plants are found at the nearest and farthest distances. The dominant species in the WLFZs are *P*. *Plebeium*, *C*. *Dactylon*, *X*. *Sibiricum and A*. *Conyzoides*. Due to the different distribution preferences of each type, the dominant species are different in each distance section.

#### Vegetation distribution as affected by slope

In the four study areas of Huangdeng1, Huangdeng2, Nuozhadu and Xiaowan (Table 3), the WLFZs with slope less than 25° account for 77.45%, 79.07%, 70.05% and 79.87% respectively, and the slopes between 25°-35° and 35°-90° account for less than 16%. Therefore, all the study areas belong to gentle slope WLFZ. The horizontal extension of the gentle slope WLFZ is relatively large. Studies have shown that the effect of the coastal buffer zone in purifying and blocking pollutants is directly proportional to its width. WLFZ as a typical coastal buffer zone, the horizontal width directly affects its purification efficiency [41, 42]. In the four study areas, the average width of the WLFZ in Huangdeng 1, Huangdeng 2, Nuozhadu and Xiaowan is about 86 meters, 34 meters, 92 meters and 146 meters respectively. It is generally believed that the 30-60m coastal buffer zone can intercept more than 50% of the sediment flowing to the river.

**Table 3:** The area, proportion and vegetation area, proportion and coverage of the WLFZ classified by slope in each study area in 2019.

Study area	Category	Slope					
		0°-8°	8°-15°	15°-25°	25°-35°	35°-60°	60°-90°
Huangdeng1	*Vegetation area*	1.76	0.77	0.45	0.28	0.17	0.18
	*WLFZ area*	3096.85	1822.62	1162.75	780.43	498.09	492.70
	*WLFZ proportion*	39.43%	23.21%	14.81%	9.94%	6.34%	6.27%
	*Vegetation proportion*	48.76%	21.24%	12.58%	7.62%	4.68%	5.11%
	Coverage rate	0.06%	0.04%	0.04%	0.04%	0.03%	0.04%
Huangdeng2	*Vegetation area*	4.59	4.84	2.84	1.80	1.04	0.79
	*WLFZ area*	421.35	426.57	315.09	184.80	84.01	38.80
	*WLFZ proportion*	28.65%	29.00%	21.42%	12.57%	5.72%	2.64%
	*Vegetation proportion*	28.86%	30.45%	17.86%	11.34%	6.55%	4.94%
	Coverage rate	1.09%	1.14%	0.90%	0.98%	1.24%	2.02%
Nuozhadu	*Vegetation area*	1526.02	978.2	1079.57	679.86	664.91	170.3
	*WLFZ area*	2137.43	1999.30	1584.51	1139.27	732.68	573.10
	*WLFZ proportion*	26.17%	24.48%	19.40%	13.95%	8.97%	7.02%
	*Vegetation proportion*	29.93%	19.18%	21.17%	13.33%	13.04%	3.34%
	Coverage rate	71.40%	48.93%	68.13%	59.68%	90.75%	29.72%
Xiaowan	*Vegetation area*	2178.51	2753.80	2127.61	764.28	378.45	59.21
	*WLFZ area*	3747.42	6473.32	4282.38	2264.39	1033.61	356.11
	*WLFZ proportion*	20.64%	35.65%	23.58%	12.47%	5.69%	1.96%
	*Vegetation proportion*	26.37%	33.33%	25.75%	9.25%	4.58%	0.72%
	Coverage rate	58.13%	42.54%	49.68%	33.75%	36.61%	16.63%

**Fig 9:**
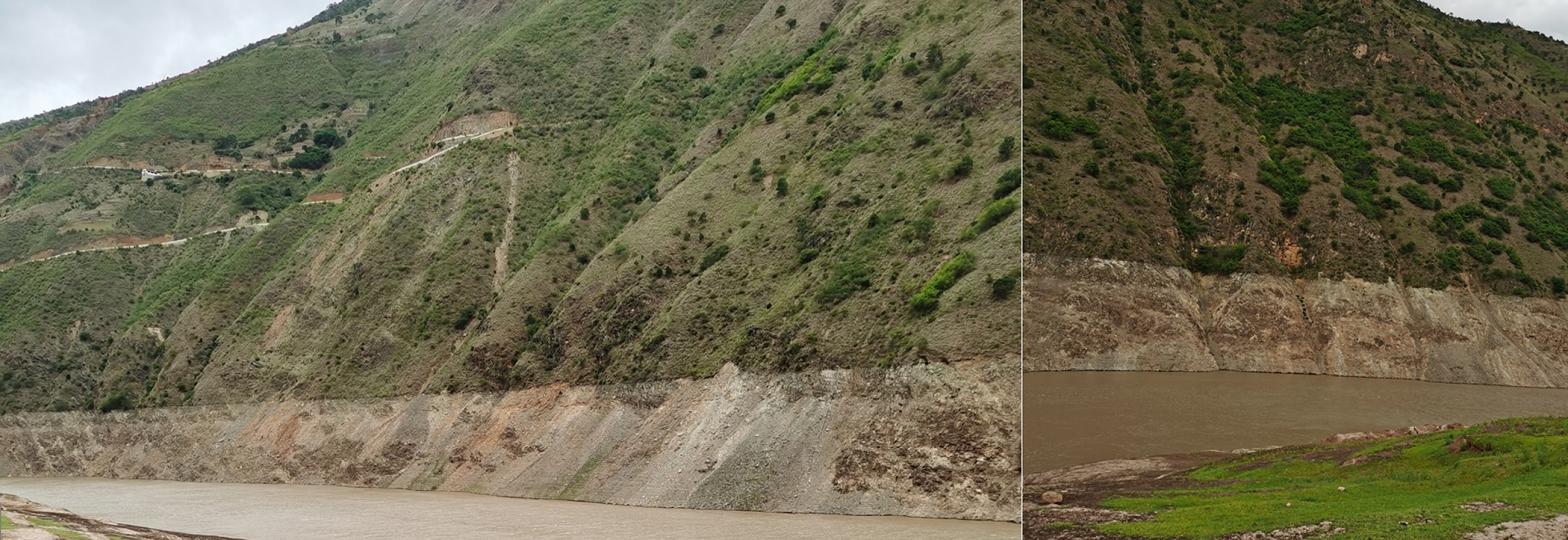
Steep-slope WLFZs of Lantsang River.

In each study area, more than 70% of the vegetation is distributed in the slope less than 25° of the zone. This is partly due to the fact that each study area belongs to a gentle slope zone, and more than 70% of the areas in the zone have a slope less than 25°. On the other hand, existing studies have shown that the erosion zone with a slope greater than 25° is affected by runoff and water level changes [43, 44]. The soil is more eroded and the bedrock is exposed, making it difficult for plants to grow. The observations along the river also confirm this point (Fig 9). Steep-slope WLFZs usually have thin soil layers, mostly exposed lithological surfaces and less vegetation coverage. Relatively speaking, gentle slopes are more suitable for vegetation growth. In the WLFZ of each study area, less than 30% of the vegetation is distributed in areas with a slope greater than 25°. According to the overlay analysis of the vegetation classification map and the slope classification map and on-site observation, the vegetation type growing in the area with a slope of 60°-90° is mainly *C*. *Dactylon*, *P*. *Plebeium*, *X*. *Sibiricum and A*. *Conyzoides*.

#### Vegetation distribution as affected by aspect

**Fig 10:**
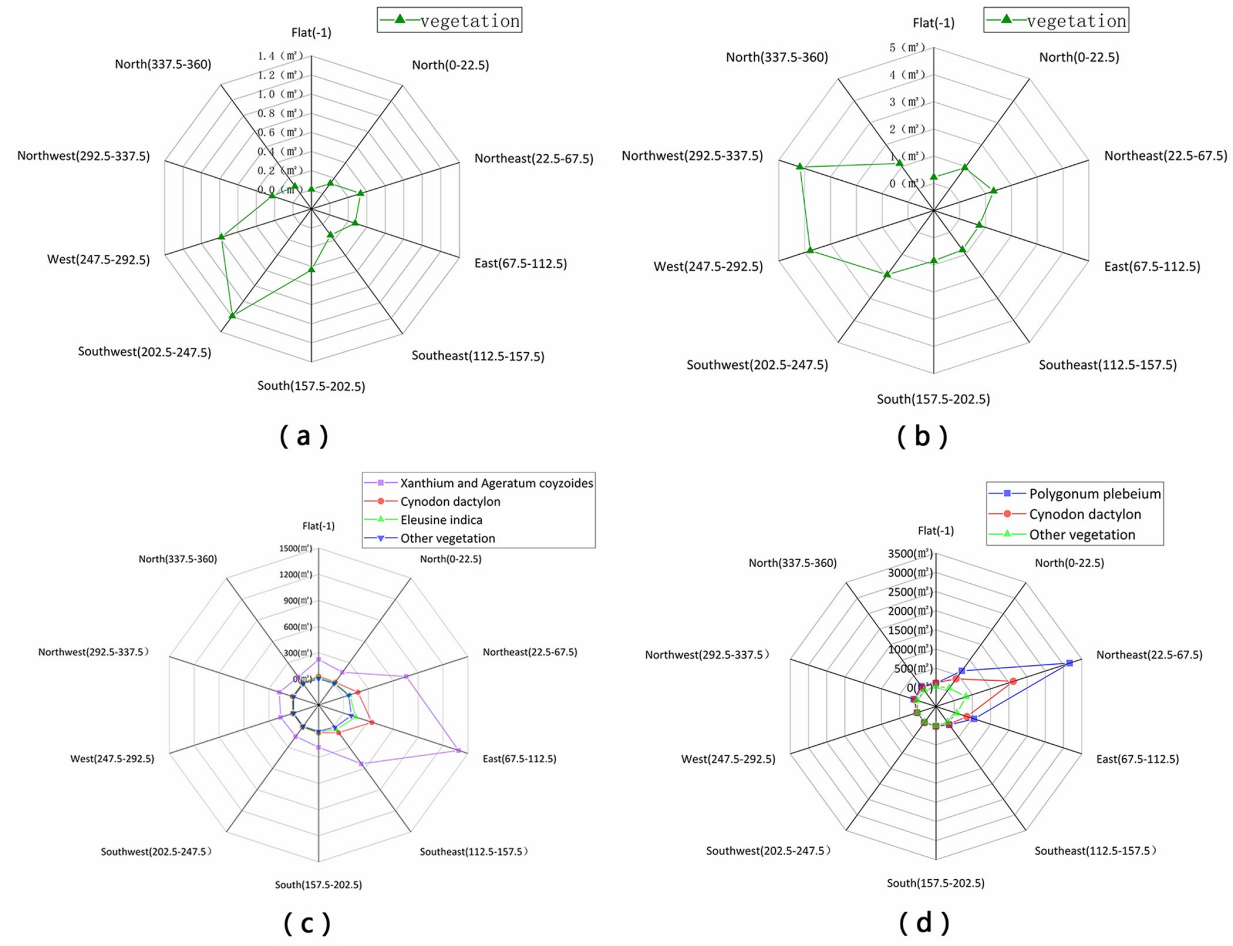
Vegetation distribution area as a function of aspect in the WLFZ of (a) Huangdeng study area 1, (b) Huangdeng study area 2, the (c) Nuozhadu study area, and the (d) Xiaowan study area.

In Huangdeng Reservoir study area 1 (Fig 10(A)), 33.09% of the vegetation in the WLFZ is distributed in the southwest, followed by 21.65% in the west, 12.12% in the south, with a small amount of vegetation in other directions. In study area 2 (Fig 10(B)), the vegetation is mainly distributed in the northwest, west and southwest, with vegetation area proportions of 26.29%, 23.78% and 12.09%, respectively. In addition, there is a small amount of vegetation in other directions.

In the study area of Nuozhadu Reservoir (Fig 10(C)), the vegetation is mainly distributed in the east, accounting for 39.03%, followed by the northeast and southeast, accounting for 21.08% and 13.45%, respectively. The amount of vegetation in the other aspects is very small, and the vegetation type is almost entirely *X*. *Sibiricum–A*. *Conyzoides* groups with a relatively uniform distribution, while *C*. *Dactylon*, *E*. *Indica* and other vegetation hardly survive.

As shown in Fig 10(D), the distribution characteristics of all kinds of vegetation in the Xiaowan Reservoir study area are relatively consistent, and the directivity is very obvious, where most plants are concentrated in the northeast direction, and the vegetation area reaches 5200 m^2^, accounting for 61.96%, followed by the east and north, with vegetation proportions of 11.69% and 13.74%, respectively, while in other directions, vegetation is rare.

As the aspect of the study area is relatively uniform, most of the vegetation has an obvious main direction and similar side directions, where the aspect of vegetation distribution is relatively consistent with that of the experimental areas themselves. The main aspects of vegetation in the four study areas cover almost all directions, indicating that vegetation in the WLFZs shows no obvious dependence on aspect.

## Discussion and conclusions

### Vegetation in the falling zone of Lantsang River Reservoir

Vegetation restoration is a long process, and its coverage and species diversity are related to the age of the WLFZ and the length of the different plant growth periods. The WLFZ in the Huangdeng Reservoir area has only just formed, and the vegetation therein has not yet experienced a relatively stable growth period. As a result, the vegetation coverage in the WLFZ is less than 2%, and there are only 1–2 vegetation species. Thus, there is little species diversity and richness, and the ecosystem is relatively fragile. Nuozhadu and Xiaowan Reservoirs have both experienced more than five growth cycles of their WLFZs, and their vegetation coverage rates have reached 45.5% and 62.44%, respectively. In each of these reservoirs, there are 7–8 vegetation types. Thus, they both have considerably more vegetation coverage and species diversity than Huangdeng Reservoir. However, when comparing the Nuozhadu and Xiaowan study areas, it was found that although the WLFZ formed in Xiaowan Reservoir three years earlier than in Nuozhadu Reservoir, the vegetation coverage rate of the Xiaowan study area was 16.94% smaller than in the Nuozhadu study area. In the Nuozhadu study area, *X*. *Sibiricum–A*. *Conyzoides* mostly exist in the form of associations, accounting for 75.03% of the vegetation in the WLFZ. *X*. *Sibiricum–A*. *Conyzoides* form a complex community structure with different plant heights, indicating that the promoting effect between them is obviously greater than any competition effect, which allows them to steadily occupy the suitable habitat and become the dominant species in Nuozhadu study area. Secondly, the Xiaowan Reservoir area is located in a typical section of the “dry-hot valley” of Lantsang River, where the site conditions for the survival of vegetation are relatively poor. Therefore, in general, vegetation restoration in a WLFZ is affected by many factors, including not only reservoir age, growth period and other time factors, but also topographical conditions, climate, population structure and the interaction among species.

Due to the periodic change to the water level caused by reservoir operation, most of the plants in the WLFZ of the Lantsang River Reservoir area are annual or perennial herbs. There are usually no more than three dominant species in the WLFZ of each reservoir, and one species or association is absolutely dominant. There are differences in the dominant species of vegetation in the WLFZs of different reservoir areas. On the whole, *X*. *Sibiricum–A*. *Conyzoides*, *P*. *Plebeiu*, *C*. *Dactylon* and *E*. *Indica* are the dominant plants in the WLFZs of Lantsang River, among which *C*. *Dactylon* is the most common dominant species in each reservoir area and a pioneer vegetation type in the WLFZ of Huangdeng Reservoir, which has many creeping stems with strong climbing and adhesion ability. With an increase of WLFZ age, an increase in the amount of vegetation types and an intensification of species competition, the pioneer advantage of *C*. *Dactylon* was gradually replaced by *P*. *Plebeiu* or *X*. *Sibiricum–A*. *Conyzoides* groups. These caused it to become the second most dominant species. These results provide a good reference for artificial restoration activities; for example, *C*. *Dactylon* can be considered as a priority species for new reservoir WLFZs, while other dominant species, such as *X*. *Sibiricum–A*. *Conyzoides*, *P*. *Plebeium* and *E*.*Indica*, can be considered for WLFZs that are older than five years. In addition, *X*. *Sibiricum–A*. *Conyzoides* steadily occupy the Nuozhadu WLFZ in a group-wise manner, which may imply that two mutually reinforcing plants are more stable than one plant.

Next, due to the special formation process of a WLFZ, flooding is one of the main factors affecting vegetation restoration therein, while elevation is an indirect consequence of the flooding situation. At different elevations in a WLFZ, the flooding time, intensity and frequency are all different. In the WLFZ of Lantsang River Reservoir, the vegetation coverage increases first and then decreases with elevation; that is, vegetation coverage in the WLFZ follows the pattern: lower part < upper part < the middle part. The results show that vegetation coverage is negatively related to the flooding time. In the lower parts of the WLFZ, the vegetation coverage is almost zero due to the longest submergence times, irregularly fluctuating water levels and water erosion. Any plants that can survive possess a rather strong ability to withstand submergence.

There is an obvious “dry-hot valley” phenomenon in the middle and upper reaches of Lantsang River, which is a valley belt with two basic properties of dry and hot, with high temperatures, low humidity, rich light and heat resources, hot climate and little rain; hence, the growth of vegetation is strongly dependent on water. Slope affects surface runoff and soil erosion, reflecting the ability of water and soil conservation. Gentle slopes within 25° are more suitable for vegetation growth. However, steep slopes above 25° are difficult to adapt to plant growth due to water erosion, thin soil and high bare rock rate. In order to prevent large-scale soil flow, engineering revetment is usually needed to assist.

In terms of the distance to the nearest water body, the type of dominant species is different in different distance sections. On the whole, vegetation coverage is the highest in the middle-range distance section from the nearest water body, which gradually decreases in the nearest and farthest distance sections, especially in the latter. This is consistent with the elevation distribution characteristics of vegetation. The upper parts of a WLFZ endure long periods of water recession. These parts are farthest from the water body, and the most stressed by seasonal drought and high temperatures. Thus, vegetation coverage in these regions is far lower than that in the middle of the WLFZ. Moreover, any vegetation growing in the upper parts has the characteristics of heat resistance, drought resistance and strong water retention.

Next, the aspect of vegetation distribution is relatively consistent with that of the study area itself. In the four study areas, the main aspects of the vegetation cover almost all directions, and the vegetation does not show an obvious aspect preference.

In the study areas of Xiaowan and Nuozhadu Reservoirs, it can be seen that there are differences in the distributions of different vegetation types, which show different topographic preferences. *C*. *Dactylon* is the second most dominant species in both study areas, and its range of suitable habitat is, which depends on topographical factors, evenly distributed. Meanwhile, the most dominant species shows a large range of changes, where the second highest value of coverage is approximately 50% of the peak value. In addition, in the elevation map and the map of distance to the nearest water body, the geographic distribution locations corresponding to the peak coverage of *C*. *Dactylon* are lower than those of the most dominant species. Other vegetation is mostly distributed in the upper and the lower parts of the WLFZ. This is because the suitable growing area in the middle of the WLFZ is occupied by dominant species. Other vegetation contains several types, but each type has rare quantities, scattered distributions, small plants, which limits their competitiveness with other plants.

### Utility of UAV for monitoring WLFZ vegetation

At present, statistical sampling surveys based on sample plots are still the main means for studying vegetation in WLFZs. However, such surveys have poor accessibility and are easily limited by complex topographies. In the Lantsang River Reservoir area, for example, many places are impossible to reach and sample. Moreover, sample plots cover only a small area, with long investigation periods, difficult data updates and high field labor intensity.

Remote-sensing technology is not limited by region, which allows for research from point sampling to surface sampling, and it allows for the realization of in-situ monitoring without destroying vegetation because of its non-contact characteristic. However, satellite remote sensing is unsuitable for vegetation investigation in WLFZs due to its limited resolution. Indeed, vegetation in WLFZs is mainly herbaceous, which is usually difficult to distinguish in satellite images, even with sub-meter resolution.

Instead, UAV remote sensing can yield high-resolution data from closer locations to meet the needs of vegetation identification in WLFZs. Moreover, compared with satellite remote sensing, which is easily limited by cloud cover and the revisit period, UAV remote sensing is cheaper and more flexible. UAV low-altitude remote sensing provides an effective to conduct ecological research on WLFZs, which can monitor vegetation dynamics in response, timeliness and at low cost, as well as providing appropriate spatial and temporal resolution data.

### Recommendations for vegetation restoration in WLFZs

Artificially promoting vegetation colonization and the growth of stable species groups can help promote their natural restoration and succession abilities in WLFZs, which is of great significance for WLFZ ecosystem restoration and the sustainable development of hydropower projects. At present, there are very few studies regarding vegetation in the WLFZs of the cascading reservoirs of Lantsang River, and the effects of blind restoration are often not ideal. This constitutes a waste of financial resources, labor and material resources. According to the research results of this paper, we suggest that gentle slopes (0–25°) in the WLFZ should be selected as a priority for the restoration of vegetation in the cascading reservoirs of Lantsang River. Then, according to the test results, the restoration should proceed in a step-by-step manner, starting with planting on the gentle slopes as a test area, so as to lay a foundation for the ecological restoration of the WLFZ in the “dry-hot valley”.

For WLFZs younger than three years, it is advisable to plant some pioneer species, such as *C*. *Dactylon*, in the middle parts of the WLFZ and on gentle slopes during artificial ecological restoration. Due to the strong influence of human activities in the early stage of WLFZ creation, the seeds of *C*. *Dactylon* can be easily spread. This can rapidly expand the overall plant population. Indeed, *C*. *Dactylon* can adapt well to the habitat of WLFZs in young reservoirs. For the WLFZs aged 3–10 years, simply planting some pioneer species is not enough to achieve good restoration of the WLFZ; instead, it is appropriate to increase the number of types of restored species. For example, in the middle of a WLFZ, it is appropriate to plant *P*. *Plebeium*, *E*. *Indica*, *X*. *Sibiricum–A*. *Conyzoides* and *C*. *Dactylon* from top to bottom. The uppermost and lower parts of the WLFZ can be planted with *D*. *Sanguinalis*, *E*. *Indica*, *E*. *Hyemale*, *R*. *Indica*, *C*. *Hederacea*, *S*. *plebeian*, *Ch*. *Ambrosioides* and so on, which can be planted in cascades, thus forming a complex community structure of plants at different heights. As such, they have the potential to form a stable ecosystem. For WLFZs older than 10 years, it may be appropriate to add some sub-shrub and shrub species, but further research is still needed to verify this.
